# AAV-mediated gene therapies for glaucoma and uveitis: are we there yet?

**DOI:** 10.1017/erm.2024.4

**Published:** 2024-04-15

**Authors:** Brenda Castro, Jason C. Steel, Christopher J. Layton

**Affiliations:** 1 LVF Ophthalmology Research Centre, Translational Research Institute, Brisbane, Australia; 2 Faculty of Medicine, Greenslopes Clinical School, The University of Queensland, Brisbane, Australia; 3 School of Health, Medical and Applied Sciences, Central Queensland University, Rockhampton, Australia

**Keywords:** AAV, degeneration, eye-disease, glaucoma, gene-therapy, inflammation, preclinical, retina, uveitis, vector

## Abstract

Glaucoma and uveitis are non-vascular ocular diseases which are among the leading causes of blindness and visual loss. These conditions have distinct characteristics and mechanisms but share a multifactorial and complex nature, making their management challenging and burdensome for patients and clinicians. Furthermore, the lack of symptoms in the early stages of glaucoma and the diverse aetiology of uveitis hinder timely and accurate diagnoses, which are a cause of poor visual outcomes under both conditions. Although current treatment is effective in most cases, it is often associated with low patient adherence and adverse events, which directly impact the overall therapeutic success. Therefore, long-lasting alternatives with improved safety and efficacy are needed. Gene therapy, particularly utilising adeno-associated virus (AAV) vectors, has emerged as a promising approach to address unmet needs in these diseases. Engineered capsids with enhanced tropism and lower immunogenicity have been proposed, along with constructs designed for targeted and controlled expression. Additionally, several pathways implicated in the pathogenesis of these conditions have been targeted with single or multigene expression cassettes, gene editing and silencing approaches. This review discusses strategies employed in AAV-based gene therapies for glaucoma and non-infectious uveitis and provides an overview of current progress and future directions.

## Graphical abstract

**Figure:**
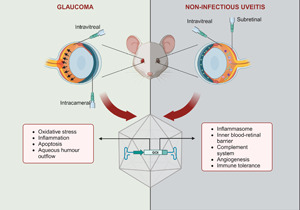

## Introduction

Glaucoma and uveitis are non-vascular ocular diseases which are among the leading causes of blindness and visual loss (Ref. 1). These chronic conditions pose a significant social and economic burden resulting from their prevalence and impact on patients' mental health, independence, work productivity and career, in addition to treatment costs. Hence, reduced quality of life is often experienced by affected patients, which are usually in the working age group (Refs 2, 3, 4, 5). Both conditions encompass a complex, multifactorial group of entities, with the clinical presentation of progressive degeneration of retinal ganglion cells (RGCs) or intraocular inflammation being the basis of diagnosis (Refs 2, 5). The exact pathogenesis of both diseases is not entirely understood, but it is known that genetic, environmental and immunological factors play important roles (Refs 6, 7, 8).

Achieving good outcomes in patients with glaucoma and the non-infectious form of uveitis may be challenging for clinicians because of the complex nature of these diseases and delayed definitive diagnosis (Refs 9, 10). Current management of glaucoma includes surgical and pharmacological interventions, both aiming at reducing intraocular pressure (IOP), the primary and only modifiable risk factor associated with the disease, a strategy that has not changed in 100 years (Ref. 2). Thus, IOP lowering to a target range by instilling eye drops from various pharmacological classes or performing laser therapy on the trabecular meshwork (TM) is the initial approach (Ref. 7). However, IOP control alone is insufficient to halt glaucoma progression in some patients, which, along with high levels of non-compliance, can lead to therapeutic failure and potentially vision loss (Refs 11, 12). Therefore, novel IOP-lowering approaches less dependent upon patient compliance and therapeutic options targeting non-IOP-related risk factors are needed to improve management of glaucoma (Refs 9, 11). Similarly, the standard of care for uveitis has remained the same since the 1950s and relies on immunosuppression by corticosteroids (CS) administered by multiple routes (Ref. 5). Unfortunately, CS are sometimes ineffective and often associated with severe local and systemic side effects, especially in long-term treatment, and this limits their clinical utility. These limitations restrict the use of CS and highlight the need for alternative therapies with better safety profiles and proven efficacy (Refs 13, 14).

Extensive research has been conducted to improve understanding of underlying mechanisms driving these chronic, difficult to treat ophthalmic conditions, and several novel therapeutic approaches have been explored. These strategies include novel agents, nanocarriers, extended-release devices and gene- and cell-based therapies, each with unique advantages and relevance to the field, which have been reviewed elsewhere (Refs 4, 10, 11, 12, 15, 16, 17, 18, 19, 20, 21, 22, 23, 24, 25).

Gene therapy has emerged as a promising approach for treating various acquired ocular disorders in addition to its established role in genetic ophthalmic disease. Adeno-associated virus (AAV) is the leading vector in ocular gene delivery because of its relatively low immunogenicity, ability to transduce a wide range of cells and versatility (Ref. 26). Although other approaches can undoubtedly bring benefits to glaucoma and uveitis patients, AAV stands out as a promising technology given the possibility of optimisation of the delivery and expression methods, including regulation, targeting, specificity and multi-acting, in addition to a scalable manufacturing process (Refs 27, 28). These distinctive features underscore the potential of AAV to address challenges associated with the management of prevalent, chronic, multifactorial and complex disorders (Ref. 27).

Recent findings from clinical trials involving AAV-based gene therapies for acquired vascular diseases offer promising insights into the potential extension of this technology to non-vascular ophthalmic conditions, including uveitis and glaucoma. In fact, preclinical studies have reported positive outcomes with constructs targeting multiple mechanisms, including neuroprotection, immunomodulation, oxidative stress or aqueous humour outflow in glaucoma, and the complement system, the inflammasome and immune tolerance in uveitis. Optimised designs, such as cell-specific promoters, multigenic cassettes and inducible systems, have been explored, in addition to gene editing, gene silencing and capsid engineering technology (Refs 29, 30, 31, 32, 33). Gene therapies encoding anti-inflammatory cytokines or peptides have also been proposed for both diseases (Refs 34, 35, 36, 37, 38).

Unfortunately, clinical translation has yet to be achieved despite the successful results observed in animal models. Accomplishing this is made more challenging because, unlike incurable genetic eye diseases, any therapy for these conditions must be evaluated against current standard-of-care treatments. Hence, their acceptance and broad adoption will only occur if compelling evidence of their advantages over existing solutions is seen (Ref. 39).

Therefore, it is important to discuss therapeutic and delivery strategies reported in preclinical studies for glaucoma and uveitis, with their respective benefits, limitations and experimental strategy to pave the way to the development of therapies with improved prospects of advancing to clinical studies. Hence, this review provides an overview of AAV-based strategies which have reached preclinical animal or human testing in these multifactorial chronic diseases. We explore therapeutic and delivery approaches such as vector modification, expression cassette optimisation and gene of interest application. We delve into the potential limitations of current therapeutic approaches and discuss the ongoing challenges associated with gene therapy for these chronic, partially treatable ocular diseases.

## Glaucoma

### Background and prevalence

Glaucoma is a complex group of optic neuropathies characterised by progressive degeneration of RGCs and nerve fibre layers and damage to the optic nerve, resulting in irreversible vision loss (Refs 2, 21). Glaucoma was the leading cause of irreversible blindness globally in 2020 and is estimated to be affecting 111.8 million people in 2040 (Refs 1, 40). The prevalence of glaucoma varies by geographic area and seems to rise as the population ages, posing a significant financial and clinical burden, which increases with the disease severity (Refs 41, 42). Different genetic and environmental risk factors have been described, including elevated IOP, older age, African background, family history, high myopia, vascular disease and obesity (Refs 21, 23, 43, 44, 45). Glaucoma can be classified as primary if idiopathic (unknown cause) or secondary when an underlying condition or event is identified (Ref. 7), or according to the anatomy of the iridocorneal angle as open-angle or angle-closure. Secondary types of glaucoma include pigmentary, congenital, uveitic, traumatic, neovascular and exfoliative (Refs 23, 46, 47). Primary open angle is the most common type of glaucoma. Chronic glaucoma often progresses asymptomatic until substantial RGC degeneration has occurred at advanced stages (Ref. 48). The visual impairment at this stage can manifest as blurriness, blind spots, impaired contrast and colour perception and loss of peripheral visual acuity. These clinical symptoms directly affect patients' quality of life by limiting their mobility and ability to perform daily activities, such as driving and reading. Early detection of glaucoma is essential to save patients' sight and improve prognosis (Refs 48, 49).

### Pathogenesis and management

The disease pathogenesis is complex and has not been completely elucidated. However, elevated IOP is related to the loss of RGCs and their axons, along with excavation of the optic nerve head, which is present in all forms of glaucoma (Refs 7, 23). Elevation of the IOP, in turn, happens as a result of an imbalance between aqueous humour secretion and drainage, with the latter occurring either through the TM and Schlemm's canal or via the uveoscleral outflow pathway (Refs 43, 48). Yet, as people with IOP at a normal range can also develop the disease (‘normal tension glaucoma’), other mechanisms are implicated in the disease pathogenesis (Ref. 48). Therefore, the complex mechanism driving neurodegeneration in glaucoma seems to involve mechanical, vascular, genetic and immunological factors (Ref. 7). Furthermore, oxidative stress, ischaemia, deficiency of neurotrophic factors, glial activation and excitotoxicity have been suggested as important processes in glaucoma development and represent important targets for neuroprotective therapies (Refs 24, 50).

Current management of glaucoma aims to lower the IOP at a target level and thereby slow degeneration, and this is achieved with topical hypotensive agents, laser therapy and surgery (Ref. 43). However, low adherence to a chronic treatment involving multiple applications, encouraged by the absence of symptoms at even moderately late stages of the disease, and aggravated by incorrect administration of eye drops, contributes to treatment failure (Refs 11, 21). In some patients, the individualised target IOP is not achieved with therapeutic interventions with acceptable side effects, resulting in continued disease progression and worsening visual impairment, thereby impacting the quality of life. In these cases, laser trabeculoplasty or incisional surgical procedures may be indicated (Refs 48, 51). Also, since IOP control alone is not enough to prevent glaucoma progression in some patients, investigation of additional therapies is needed (Refs 12, 24). In this context, gene therapies may benefit glaucoma patients by either targeting genes associated with the condition or inducing the expression of factors which influence the pathogenesis (Refs 12, 20). Indeed, as mentioned, AAV vectors have been widely employed to deliver genes encoding molecules with neuroprotective, antioxidant, antiapoptotic or anti-inflammatory effects, as well as those affecting the aqueous humour outflow (Table 1).

**Table 1.** Preclinical studies using AAV-mediated gene therapies in models of glaucoma

Therapeutic gene	Proposed pathway/mechanism	Vector	Promoter	Administration route	Dose	Timing of treatment	Disease model/specie	Ref.
BDNF and TrkB	BDNF/TrkB signalling *–* neuroprotection	AAV2	CAG	Intravitreal	1 × 10^9^ (rats); 1 × 10^10^ vg (rats and mice)	Before disease induction	Optic nerve crush/mice; laser-induced IOP elevation/rats	32
BDNF	BDNF/TrkB signalling *–* neuroprotection	AAV2	CMV	Intravitreal	2 × 10^10^ vg	Three weeks before disease induction	Microbead-induced trabecular occlusion/rats	56
Brn3b	Bcl-2-mediated antiapoptotic pathways – neuroprotection	AAV2	CMV and hSyn	Intravitreal	1 × 10^9^–3.5 × 10^9^ vg	One week after disease induction	Morrison's ocular hypertension model/rats	33, 59
Bcl-xL	Bcl-xL-mediated antiapoptotic pathways – neuroprotection	AAV2	Pgk	Intravitreal	1.7 × 10^10^ vg	One month before disease induction	Optic nerve crush and DBA/2J mice	60
MAX	MYC/MXD axis – neuroprotection	AAV2	CAG	Intravitreal	3 × 10^9^ vg	Four weeks before disease induction	Optic nerve crush and Limbal plexus cautery ocular hypertension/rats	30
Nrf2 and BDNF	Nrf2 signalling – neuroprotection	AAV2	MCP-1	Intravitreal	2 × 10^9^ vg	Not reported	Optic nerve crush/mice	62
SOD2	Antioxidative	AAV2	CAG	Intravitreal	2 × 10^9^ vg	Twenty-one days before disease induction	Laser photocoagulation to the trabecular meshwork and episcleral veins/rats	29
ABCA1	Annexin 1-mediated signalling – antiapoptotic	AAV8	CMV	Intravitreal	1.5 × 10^9^ vg	Ten days before disease induction	Ischaemia/reperfusion/mice	31
C3	Rho-signalling – neuroprotection	scAAV2	CAG	Intravitreal	2.5 × 10^9^ vg	Seven days before disease induction	Ischaemia/reperfusion/rats	65
CRISPR-Cas9 for aquaporin 1	Aquaporin 1 disruption – aqueous humour outflow	AAVsh10	CMV	Intravitreal	2 × 10^10^ vg	One week after disease induction	Corticosteroid- and microbead-induced ocular hypertension/mice	69
MMP-3	Aqueous humour outflow	AAV2/9	Tetracycline- inducible promoter and CMV	Intracameral	3 × 10^9^ vg	Two weeks before dexamethasone and in 4 months old mice	Dexamethasone-induced ocular hypertension and transgenic myocilin model/mice	72

AAV, adeno-associated virus; vg, vector genome; ABCA1, ATP-binding cassette transporter A1; Bcl-2, B cell leukaemia/lymphoma 2; Bcl-xL, B cell lymphoma extra-large; BDNF, brain-derived neurotrophic factor; Brn3b, brain-specific homoeobox/POU domain protein 3b; C3, C3 exoenzyme transferase; CAG, cytomegalovirus enhancer – chicken beta-actin hybrid promoter; CMV, cytomegalovirus; CRISPR, clustered regularly interspaced short palindromic repeats; hSyn, human synapsin 1 gene promoter; IOP, intraocular pressure; MAX, MYC-associated protein X; MCP-1, monocyte chemoattractant protein-1; MMP-3, matrix metalloproteinase-3; Nrf2, nuclear factor erythroid 2-related factor 2; Pgk, phosphoglycerokinase; scAAV2, self-complementary AAV2; SOD2, superoxide dismutase 2; TrkB, tropomyosin-related receptor kinase-B.

### Gene therapy approaches

#### AAV-mediated gene therapies targeting neuroprotection

Gene therapies providing neuroprotection for RGCs can either target natural survival pathways or prevent RGCs from progressing to cell death (Ref. 30). The first approach can be accomplished by overexpressing neurotrophic factors, such as the brain-derived neurotrophic factor (BDNF), which has shown therapeutic potential in multiple animal models of glaucoma (Refs 32, 52, 53, 54, 55, 56). Although BDNF overexpression has indeed led to neuroprotection in these studies, its long-term effect, desired in therapies for chronic diseases, is hindered partly because of the downregulation of its receptor, which seems to occur in response to the overexpression (Ref. 32). Thus, to overcome it, Osborne *et al*. developed a dual-acting AAV2 system co-expressing BDNF and its receptor, the tropomyosin-related receptor kinase-B (TrkB) (Ref. 32). The therapy was injected intravitreally in mice and rats before optic nerve crush and laser-induced IOP elevation models, respectively. Expression of both proteins for up to 6 months was reported, along with RGC survival and functional improvements, with better neuroprotection seen with the dual system compared with each transgene alone. The expression of proteins involved in BDNF/TrkB signalling-mediated pathways was also demonstrated. Yet, more studies should be conducted to ensure the safety of this therapy since elevated expression of BDNF/TrkB has been associated with increased glutamate excitotoxicity, which may impair neuronal homoeostasis (Ref. 57).

Conversely, Wojcik-Gryciuk *et al*. argue that there is a correlation between BDNF overexpression and long-term TrkB downregulation (Ref. 56). The authors observed that moderate overexpression of BDNF mediated by AAV2 vectors did not result in downregulation of TrkB and led to long-term neuroprotection (6 weeks after disease induction). The outcome was achieved with intravitreal delivery of the therapy given before induction of the trabecular occlusion model of glaucoma. The authors proposed control of BDNF levels as the critical factor for long-term responsiveness of RGCs and maintenance of TrkB levels. However, the dose utilised to achieve this moderate expression was two times higher than the high dose used by Osborne *et al*. (Ref. 32), which led to improved neuroprotection when BDNF was co-expressed with the receptor, although different animal models and time-points were assessed. Still, knowing that BDNF can also activate signalling pathways promoting cell death and that both BDNF and TrkB are expressed in malignant gliomas, a controlled expression of these proteins may be the best strategy to enable their safe practical application in glaucoma gene therapies and enable modulation of the expression levels (Ref. 58).

An alternative approach targeting neuroprotection was investigated by Stankowska *et al*. (Ref. 59) and Phatak *et al*. (Ref. 33), who employed AAV2 vectors packaging the brain-specific homoeobox/POU domain protein 3b (Brn3b), a transcription factor that controls RGC development [(Refs 33, 59). Stankowska *et al*. (Ref. 59) proposed a neuron-specific strategy by employing the human synapsin 1 promoter (hSyn). The study reported neuroprotective effects in axons of the optic nerve and RGCs, accompanied by partially restored visual function in animals subjected to Morrison's model of glaucoma. The same animal model was used in Phatak *et al*. to assess the efficacy of a constitutively expressed construct which was intravitreally injected after disease induction (Ref. 33). Protection of RGCs and their axons was found in rats treated with AAV2-Brn3b, and it was attributed to the upregulation of B cell leukaemia/lymphoma 2 (Bcl-2) (Ref. 33). The B cell lymphoma extra-large (Bcl-xL) antiapoptotic protein was also upregulated in Brn3b-treated animals, although not statistically different from green fluorescent protein (GFP) controls. Bcl-xL is a member of the Bcl-2 family predominantly expressed in retinas and plays an essential role in RGC survival (Ref. 33). Donahue *et al*. reported the efficacy of Bcl-xL overexpression through AAV2 vectors in two models of glaucoma, the optic nerve crush and DBA/2J mice (Ref. 60). The gene therapy contained a neuron-specific promoter, the phosphoglycerokinase, for RGC-targeted expression of the antiapoptotic protein. The formulation was injected intravitreally just before DBA/2J mice exhibited elevated IOP. Although this therapy did not inhibit IOP increase, Bcl-xL protected RGCs from degeneration in both models. Despite the positive outcome, the authors drew attention to potential safety concerns in long-term overexpression of antiapoptotic proteins, owing to their possible effect on oncogenesis, as discussed elsewhere (Ref. 30). Therefore, vector optimisation incorporating elements for a targeted and regulated expression may be the most suitable approach and increase the chance of clinical translation of gene therapies encoding antiapoptotic proteins and other therapeutic molecules whose continuous or widespread overexpression might be harmful.

Lani-Louzada *et al*. sought to avoid potential teratogenic side effects associated with antiapoptotic proteins and neurotrophic factors by utilising a tumour suppressor gene, the MYC-associated protein X (MAX) (Ref. 30). MAX was packaged into AAV2 vectors and intravitreally delivered to rats before optic nerve crush or the limbal plexus cautery models of glaucoma (Ref. 30). The therapy did not affect IOP dynamics and prevented RGC degeneration in both models, with no adverse events reported. However, further studies should assess systemic transgene expression and address potential side effects, given the ubiquitous nature of MAX and the promoter used, in addition to the likelihood of systemic exposure after intravitreal injection and the broad tropism of AAV2 (Ref. 61). Nevertheless, positive findings reported with MAX encourage the investigation of similar genes in neurodegenerative diseases and may expand the range of candidates for glaucoma gene therapy.

Attempts have also been made to overcome the theoretical detrimental effects of unspecific expression of neurotrophic factors by using physiologically induced promoters. Fujita *et al*. aimed to direct the expression to cells at risk of degeneration by utilising the monocyte chemoattractant protein-1 (MCP-1), an early-stress inducible promoter shown to be upregulated preceding RGC death (Ref. 62). AAV2 vectors were used to deliver an MCP-1 promoter driving the expression of either nuclear factor erythroid 2-related factor 2 (Nrf2) a regulator of multiple antioxidant genes or BDNF (Ref. 62). Intravitreal treatment with one of the two therapies in mice subjected to the optic nerve crush model resulted in decreased cell death and higher expression of RGC markers, with Nrf2 leading to better RGC neuroprotection. Comparative analysis between constitutive and MCP-1-controlled expression of Nrf2 revealed similar therapeutic effects in terms of RGC protection and contrast sensitivity, although both strategies could not improve visual acuity (Ref. 62). Since Nrf2 seems to promote RGC degeneration when constitutively expressed, Fujita *et al*. (Ref. 62) seem to have enabled safe application of Nrf2 in an injury model of glaucoma by timely and spatially modulating its expression with a stress-inducible promoter.

#### AAV-mediated gene therapies targeting oxidative stress and inflammation

Alternative gene therapies for glaucoma seek to counteract the oxidative stress and inflammation involved in the disease pathogenesis. For instance, Jiang *et al*. used AAV2 vectors encoding superoxide dismutase 2 (SOD2), an antioxidant enzyme, in rats subjected to a chronic glaucoma model induced by laser burns at the TM and episcleral veins (Ref. 29). The therapy was delivered intravitreally before disease induction, and expression of SOD2 was detected 4 weeks after laser burns. Although RGC survival and preservation of mitochondrial function were reported, these effects did not endure, and complete elimination of the oxidative stress was not achieved (Ref. 29). Still, pretreatment with AAV2-SOD2 increased the activity/expression of antioxidant enzymes and decreased retinal malondialdehide content, a marker of oxidative stress (Ref. 29). Luo *et al*. used AAV8 vectors to express a fragment of ATP-binding cassette transporter A1 (ABCA1), thought to regulate the nuclear translocation of the anti-inflammatory molecule annexin A1 (ANXA1), a process associated with induction of apoptosis in neuronal cells (Ref. 31). Intravitreal treatment in mice later subjected to the ischaemia/reperfusion model led to reduced ANXA1 nuclear translocation and prevented RGC degeneration. Interestingly, this was achieved with the expression of a fragment containing only 441 amino acids, whereas the entire length of this protein is 2261 amino acids long (Ref. 31). In fact, the authors tested two different fragments of ABCA1 and only the section containing the amino acids 903–1344 demonstrated therapeutic effects (Ref. 31). Nevertheless, this therapy was ineffective when treating the animals after disease induction, suggesting that existing cell damage could affect its efficacy, a limitation commonly observed in gene therapies for glaucoma. Still, several other targets implicated in decreasing or preventing neuroinflammation in glaucoma have been identified and may be explored in future preclinical investigations. Despite limitations, SOD2 and ABCA1 exhibited encouraging results regarding their respective therapeutic properties. Therefore, both therapies might benefit from vectors with broader tropism to achieve a long-lasting effect independent of cells highly affected during the disease course, as reported with certain engineered vectors. Notably, the AAV2.7m8 has shown effective retinal transduction following both intravitreal and subretinal delivery in degenerated mice retina, as well as in healthy explants from human donors and primates (Ref. 63).

#### AAV-mediated gene therapies targeting the aqueous humour outflow

Given the significance of aqueous humour outflow regulation on glaucoma pathogenesis, additional strategies have explored the expression of molecules involved in this process as potential gene therapies. Tan *et al*. assessed the efficacy of overexpression of C3 exoenzyme transferase (C3), a molecule which disrupts the actin cytoskeleton and cellular adhesion in TM cells by inactivating Rho, whose signalling pathway is crucial in IOP regulation (Refs 64, 65). Using a self-complementary AAV2 (scAAV2), intracameral C3 expression lowered IOP in healthy monkeys and mice despite some adverse reactions (Refs 64, 66). scAAV is a modified vector with a double-stranded genome and half-load capacity often used when targeting the anterior segment because of the reported inability of TM cells to convert AAV single-stranded DNA into a double-strand (Ref. 67).

This preliminary test assessed the intracameral administration of scAAV2-C3, but the same authors utilised the intravitreal route to investigate this therapy in rats subjected to the ischaemia/reperfusion injury model. The treatment protected retinal neuronal cells from damage and alleviated retinal thickness reduction (Ref. 65). The therapy also led to decreased expression of apoptotic markers and increased RGC survival. The outcomes were attributed to the effect of C3 on Rho GTPases involved in cell proliferation and apoptosis (Ref. 65). The multiple effects of C3 targeting different processes involved in the pathogenesis of glaucoma make it an attractive candidate for glaucoma therapy. However, questions remain regarding the safety of intravitreal scAAV2-C3 and whether transduction of TM cells and lower IOP are achieved by this route. Although intracameral administration delivers the therapeutic agent directly into the anterior chamber, drugs injected intravitreally primarily target the retina but can be eliminated through the anterior route by diffusion and using the aqueous humour turnover (Ref. 68). Furthermore, scAAV2 seems to be a suitable vector for intracameral delivery, whereas for intravitreal administration, the single-stranded vector exhibits higher transduction efficiency (Ref. 65).

Another effort to optimise cellular targeting was made by Wu *et al*., who chose AAVshH10 for a glaucoma gene therapy after an initial study showed its more efficient transduction in ciliary body non-pigmented epithelium with intravitreal injection when compared with four other serotypes (Ref. 69). Additionally, the study leveraged gene editing technology to reduce aqueous humour production by depleting aquaporins. A small Cas9 variant, the *Staphylococcus aureus*-derived CRISPR-Cas9 system was packaged into AAVshH10 vectors along with one of two short guide RNAs, both selected for their efficacy and exon location. The authors first showed that treatment with a mix of AAV formulations delivering each of the two short guide RNAs was more beneficial than each gene therapy alone. Hence, an intravitreal treatment containing 1:1 mix of the two therapies was given to mice 1 week after CS- and microbead-induced models of ocular hypertension. Although Aqp1 is present in various ocular tissues and a ubiquitous promoter was employed, selected Aqp1 disruption in the ciliary body was achieved with this combined therapy, with a reduction in IOP and protection of RGC reported in both disease models. However, decreasing aqueous humour production is a mechanic approach only, which does not affect all other pathophysiological processes that lead to glaucoma. Hence, the therapy might not have a long-term therapeutic effect or slow disease progression (Refs 20, 70). Nevertheless, combining small gene editing systems with engineered capsids brings a novel perspective on targeted gene therapy for glaucoma. It is noteworthy, however, that the application of gene editing therapies is still limited because of safety concerns regarding off-target effects and oncogenesis (Ref. 71). Still, gene editing research is quickly advancing with more clinical trials underway, which might address these limitations and ease the clinical translation of this technology.

The aqueous humour outflow and IOP homoeostasis are also affected by the activity of matrix metalloproteinases (MMPs), a family of extracellular matrix (ECM) modifying proteases expressed in the eye and most tissues (Ref. 72). O'Callaghan *et al*. explored the role of the MMP type 3 (MMP-3), a small protease that degrades the ECM, in glaucoma and developed a gene therapy consisting of AAV2/9 delivering MMP-3, under the control of the tetracycline-inducible promoter (Ref. 72). The tetracycline-inducible system in the configuration used in the study reversibly activates gene expression in the presence of doxycycline. The therapy was assessed in two murine models of glaucoma, dexamethasone-induced ocular hypertension and the transgenic mouse with myocilin Y437H mutation (Tg-MYOC^Y437H^). Intracameral injection of the therapy was given before dexamethasone administration for the first model or in 4-month-old Tg-MYOC^Y437H^ mice for the second model. Treatment with doxycycline eye drops twice daily for 2 weeks resulted in decreased IOP and signs of ECM degradation in both disease models, with no alterations in control animals. Increased outflow facility was reported in glaucomatous and control eyes, although the latter showed a higher raise. Similarly, increase in outflow facility by MMP-3 delivered by the same vector and injection route was reported in healthy non-human primates (NHPs) and human donor eyes, although with a constitutive expression system (Ref. 72). Interestingly, the authors also developed a similar gene therapy with a codon-optimised sequence of MMP-3, which increased the transgene expression in vitro (human cells) and in vivo (mice and NHPs) compared with the native form. However, only the native form was assessed in the disease models. Therefore, the approaches described by O'Callaghan *et al*. (Ref. 72) hold great promise for glaucoma therapy, considering that individualised doxycycline treatments may improve disease control in patients with different clinical settings, although it would create an added compliance burden. Furthermore, the inducible strategy is relevant given that MMPs and their inhibitors are highly expressed in other organs, and off-target effects could lead to proteolysis (Ref. 73). In addition, the increased expression levels achieved with the codon-optimised sequence may contribute to reducing vectors' dosage and improve the safety of the gene therapy should further investigation prove its efficacy in disease models.

### Current progress and future directions

**Figure 1.**
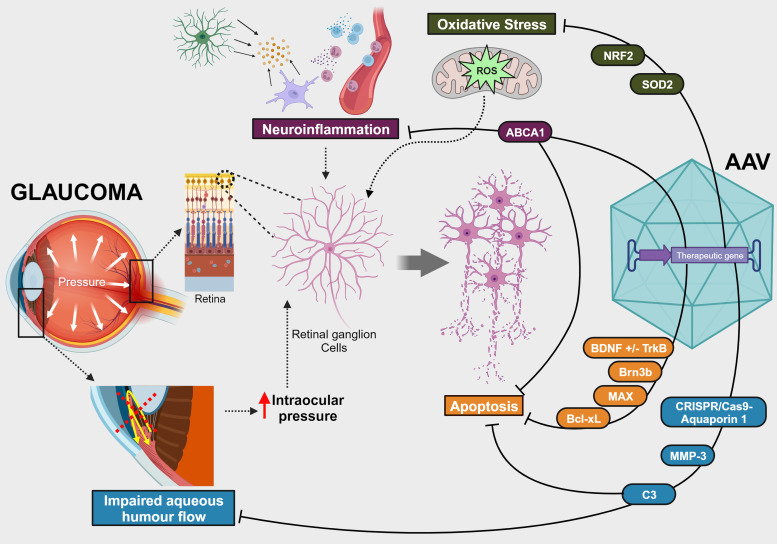
Overview of delivered genes and pathophysiological targets of recent preclinical studies with AAV-mediated gene therapies for the treatment of glaucoma. AAV, adeno-associated virus; ABCA1, ATP-binding cassette transporter A1; Bcl-xL, B cell lymphoma extra-large; BDNF, brain-derived neurotrophic factor; Brn3b, brain-specific homoeobox/POU domain protein 3b; C3, C3 exoenzyme transferase; CRISPR, clustered regularly interspaced short palindromic repeats; MAX, MYC-associated protein X; MMP-3, matrix metalloproteinase-3; Nrf2, nuclear factor erythroid 2-related factor 2; SOD2, superoxide dismutase 2; TrkB, tropomyosin-related receptor kinase-B.

Collectively, these diverse approaches (Fig. 1; Table 2) underscore the difficulty of planning and designing gene therapies for a multifactorial chronic disease with slow progression, such as glaucoma. In addition, the virtual impossibility of mirroring a complex pathogenesis and disease course in experimental models and the need for long-duration trials to accurately assess drug candidates make the development process very challenging. Nevertheless, one potential strategy to address some of these obstacles is employing gene therapies that deliver dual-acting cassettes, which can simultaneously affect multiple processes and pathways implicated in the disease pathogenesis. Additionally, engineered vectors, cell-specific promoters and inducible systems may help mitigate safety issues, given that a significant number of studies reported concerns with the overexpression of various genes of interest, emphasising the importance of carefully managing dosage, off-target effects and expression levels. Furthermore, with extensive ongoing research, an in-depth understanding of disease mechanisms and prospective targets is on track. Advances in ophthalmic imaging techniques have also allowed better monitoring of patients and may contribute to improved selection of patients and endpoints in clinical trials.

**Table 2.** Strategies and main outcomes of preclinical studies using AAV-mediated gene therapies in models of glaucoma

Vector-related strategy	Construct-related strategy	Main outcomes	Ref.
Wild-type serotype with wide retinal transduction (AAV2)	Constitutive expression and multigenic construct co-expressing BDNF and its receptor	Co-expression of both transgenes for up to 24 weeks accompanied by neuroprotection. Outperformed controls treated with AAV expressing each transgene alone	32
Wild-type serotype with wide retinal transduction (AAV2)	Constitutive expression	RGC survival and BDNF expression without downregulation of TrkB at 9 weeks after treatment and 6 weeks after disease induction	56
Wild-type serotype with wide retinal transduction (AAV2)	Neuron-specific promoter for targeted expression	RGC survival and upregulation of axonal integrity markers. Partially restored visual function	59
Wild-type serotype with wide retinal transduction (AAV2)	Constitutive expression	Increased Bcl-2 expression 3 weeks after injection. Upregulation of Bcl-xL and AKT although not significantly different from control	33
Wild-type serotype with wide retinal transduction (AAV2)	Neuron-specific promoter targeted expression	Reduction of RGC loss at 4 and 12 weeks after disease induction. Preservation of RGC and optic nerve degeneration in aged DBA/2J mice. IOP elevation was not affected	60
Wild-type serotype with wide retinal transduction (AAV2)	Constitutive expression	MAX expression 30 days after injection. Protection of RGC and improvement of functional responses. IOP elevation was not affected	30
Wild-type serotype with wide retinal transduction (AAV2)	Early-stress responsive promoter for spatial and temporal control	Effective induction of expression after injury followed by RGC survival and reduced expression of stress response markers in comparison with constitutive control. The therapy did not improve visual acuity but enhanced contrast sensitivity, similarly to constitutive control	62
Wild-type serotype with wide retinal transduction (AAV2)	Constitutive expression	SOD2 expression at 7 weeks after injection. Reduction of axon degeneration. It increased expression of antioxidative enzymes and decreased levels of an oxidative stress marker, although values were still different from healthy controls	29
Wild-type serotype with weak overall retinal transduction after intravitreal delivery (AAV8)	Constitutive expression	Upregulation of ABCA1 and reduction of annexin A1 nuclear localisation. Reduction of retinal degeneration and RGC apoptosis with pretreatment but not post-treatment	31
Self-complementary vector for faster transgene expression; wild-type serotype with wide retinal transduction (AAV2)	Constitutive expression	It increased expression of RhoA in retinas at 7 and 14 days after treatment. Prevention of retina thinning, reduction of apoptotic markers expression and promotion of RGC and neuronal cells survival	65
An engineered serotype with high expression in ciliary body non-pigmented epithelium (AAV ShH10)	Gene editing using a small CRISPR-Cas9 system; constitutive expression	Efficient aquaporin 1 disruption followed by reduced IOP and protection of RGCs at 3 weeks after treatment. Severe ocular inflammation reported in two eyes	69
Wild-type serotype with tropism to corneal endothelial cells (AAV9)	Inducible expression	Effective induction of expression with topical doxycycline. It decreased IOP in both hypertense and normotensive eyes. Promotion of extracellular matrix degradation and increased outflow facility in mice with ocular hypertension. Constitutive expression increased outflow facility in healthy primates and human explants	72

AAV, adeno-associated virus; ABCA1, ATP-binding cassette transporter A1; Bcl-2, B cell leukaemia/lymphoma 2; Bcl-xL, B cell lymphoma extra-large; BDNF, brain-derived neurotrophic factor; IOP, intraocular pressure; MAX, MYC-associated protein X; RGC, retinal ganglion cells; SOD2, superoxide dismutase 2; TrkB, tropomyosin-related receptor kinase-B; AKT, protein kinase B.

## Uveitis

### Background and prevalence

An eye is an immune-privileged organ because of multiple mechanisms, including molecular and anatomical features regulating intraocular innate and adaptive responses (Ref. 74). Yet, this highly nuanced defence can fail or become overloaded, leaving eye tissues susceptible to inflammation and infection (Refs 75, 76). Uveitis refers to inflammation of the uveal tract, composed of the iris, ciliary body and choroid, and can also affect adjacent structures. Uveitis is a heterogeneous group of sight-threatening conditions, accounting for 10–20% of preventable blindness cases in developed countries and 25% in the developing world (Refs 4, 77). It can be an isolated disease or an ocular manifestation of systemic syndromes (Ref. 78). Severe disease, inadequate disease management or lack of treatment can lead to severe vision loss and blindness because of complications including cataracts, glaucoma, vitreous debris, retinopathy and, most commonly, macular oedema (Refs 79, 80). The latter is the main condition related to vision loss in advanced uveitis and can persist or recur even after the ocular inflammation has improved or resolved (Ref. 81). Other complications seen in late stages of the disease are retinal detachment, optic disc atrophy and phthisis, a term referring to a shrunk non-functional eye (Ref. 82).

Consequently, similar to glaucoma, uveitis has a significant socioeconomic impact, particularly considering its high prevalence among young adults of working age (Ref. 4). In fact, 35% of patients with uveitis present with legal blindness or significant vision loss (Ref. 83). The standardisation of uveitis nomenclature group classifies the disease as anterior, intermediate, posterior or panuveitis, based on the primary anatomical site of inflammation. Additional descriptors include disease onset, duration and course (Ref. 84). Uveitis can be further classified clinically according to its aetiology, which includes infectious (bacterial, viral, fungal or parasitic), non-infectious and masquerade (neoplastic or non-neoplastic) (Ref. 85). Non-infectious uveitis (NIU) is the most prevalent type in developed countries and can be categorised as autoimmune, secondary to an underlying systemic condition or idiopathic (Ref. 86). This review will focus on NIU since treatment for the infectious form and masquerade syndromes aims at tackling the pathogen or the underlying condition, respectively.

Clinical symptoms of NIU can differ considerably even within the same category. Anterior uveitis may manifest with pain and redness, particularly when associated with spondyloarthritis/human leucocyte antigen (HLA) B27. It can also present with blurred vision, ocular injection, watering and sensitivity to light. In advanced stages the condition can lead to synechiae, which is when the pupil exhibits an irregular shape because of adhesions between the iris and the cornea or lens (Refs 87, 88, 89). Symptoms and signs are less evident when the inflammation involves the posterior segment. In this case, blurred vision and floaters are reported in intermediate uveitis, whereas the posterior type additionally presents vision loss and visual disturbances called dysphotopsias (Refs 82, 90). Panuveitis can manifest as a combination of all these symptoms (Ref. 88).

### Pathogenesis and management

Although the pathogenesis of NIU has not been fully elucidated, the influence of genetic and environmental risk factors has been suggested (Ref. 6). Similar to other autoimmune diseases, it is thought that an imbalance between regulatory and inflammatory mechanisms, potentially triggered by trauma or environmental factors, combined with genetic background, drives the manifestation and progression of uveitis. Additionally, the autoimmune response plays an important role in NIU pathophysiology (Refs 77, 91). The management of NIU aims to control the inflammation and achieve sustained remission in order to avoid ocular complications while minimising potential side effects associated with the therapy (Ref. 25). CS are usually employed as first-line agents for a powerful, short-term anti-inflammatory effect. Once the acute inflammation is controlled, the long-term management of uveitis is challenging and depends on several aspects, including disease severity, association with a systemic condition and patient's particularities (Ref. 92). A stepwise approach is typically employed, introducing different immunomodulatory agents, either alone or in combination, to reduce the CS burden and their attendant ocular and systemic adverse events, especially in prolonged use. Patients exhibiting a suboptimal response to the first-line agent are also subjected to a CS-sparing therapy, which is required in a significant number of cases, either for long-term treatment or because of therapeutic failure (Refs 4, 86, 93). Hence, antimetabolites, calcineurin inhibitors, biologics and less frequently alkylating agents are utilised as second- and third-line agents. However, severe side effects such as glaucoma, cataracts, liver failure and immunosuppression, along with high costs, less reliable efficacy and clinicians' unfamiliarity, limit the use of these drugs. Consequently, CS remain the mainstay therapy for uveitis (Refs 15, 94). Therefore, there is still an unmet need for therapies that offer improved safety profiles, increased efficacy and long-lasting local effects. Extensive research has been conducted, identifying new inflammatory pathways involved in uveitis and enabling the development of promising candidates, including gene therapies. Specifically, AAV-mediated gene therapies promoting local expression of anti-inflammatory factors are advantageous alternatives to current uveitis treatment (Table 3).

**Table 3.** Preclinical studies using AAV-mediated gene therapies in models of NIU

Therapeutic gene	Proposed pathway/mechanism	Vector	Promoter	Administration route	Dose	Timing of treatment	Disease model/specie	Ref.
M013	Inflammasome and NF-κB – anti-inflammatory	AAV2 (quadY-F + T-V)	smCBA	Intravitreal	3 × 10^9^ vg	One month before disease induction	EIU/mice	95
M013	Inflammasome and NF-κB – anti-inflammatory	AAV2 (quadY-F + T-V)	smCBA	Intravitreal	3 × 10^10^ vg	One month before disease induction	EAU/mice	38
shRNA silencing TLR7	TLR7 signalling – anti-inflammatory	AAV8	U6	Subretinal	4 × 10^10^ vg	On the day of disease induction	EAU induced by IRBP and adoptive transfer of IRBP-specific T cells/mice	37
Peptide derived from Nrf2	Nrf2 signalling – antioxidative and anti-inflammatory	AAV2 (quadY-F + T-V)	Chicken β-actin	Intravitreal	3 × 10^9^ vg	One month before disease induction	EIU/mice RPE-induced injury/mice	98
Three inhibitors: against VEGFA, C3b/C4b and both	Classical and alternative complement pathways and VEGF signalling – anti-inflammatory and antiangiogenic	AAV2 (sext Y-F)	Chicken β-actin	Intravitreal	7.5 × 10^8^ vg	Three weeks before disease induction	EAU/mice EIU/mice CNV/mice	36
CD59	Complement cascade – anti-inflammatory	AAV2	Chicken β-actin	Intravitreal	3.5 × 10^9^ vg	One week before disease induction	EAU/mice	104
ACE2	ACE2/Ang-(1–7)/Mas – anti-inflammatory	AAV8 (Y733F)	Chicken β-actin	Subretinal	1 × 10^8^ vg	Three weeks before disease induction	EAU/mice	112
HLA-G1 and HLA-G5	HLA-G-mediated – immune tolerance	scAAV8	JeT	Intravitreal	2.4 × 10^10^ vg	One week before disease induction	EAU/rats	34

AAV, adeno-associated virus; vg, vector genome; ACE2, angiotensin-converting enzyme 2; Ang-(1–7), angiotensin-(1–7); CNV, choroidal neovascularisation; EAU, experimental autoimmune uveitis; EIU, endotoxin-induced uveitis; HLA-G, human leucocyte antigen-G; IRBP, interphotoreceptor retinoid-binding protein; Mas, Mas receptor; NF-κB, nuclear factor kappa B; NIU, non-infectious uveitis; Nrf2, nuclear factor erythroid factor 2-related factor 2; RPE, retinal pigment epithelium; scAAV8, self-complementary AAV8; shRNA, short hairpin RNA; smCBA, truncated chimeric cytomegalovirus (CMV) chicken β-actin; TLR7, Toll-like receptor 7; VEGFA, vascular endothelial growth factor A.

### AAV-mediated gene therapies

#### Gene therapies assessed in multiple models of uveitis

Ildefonso *et al*. developed a therapy attempting to address some of these factors, consisting of a variant of AAV2 encoding a secretable and cell-penetrating form of M013, a protein from the myxoma virus with inflammasome and nuclear factor kappa B (NF-κB) inhibitory properties (Ref. 95). The M013 gene was fused to the sequence of Tat peptide from human immunodeficiency virus (HIV) to enable the secreted protein to penetrate nearby cells and exert its effect (Ref. 95). The AAV2 variant (quadY-F + T-V) contained four tyrosine-phenylalanine mutations (at positions 272, 444, 500 and 730) and one threonine-valine mutation at position 491. This serotype had previously showed efficient transduction in several retinal cells after intravitreal delivery (Ref. 95). In a mouse model of endotoxin-induced uveitis (EIU), intravitreal pre-treatment with the therapy led to reduced inflammatory infiltrate and lower levels of interleukin 1β (IL-1β) in the vitreous compared with sham controls. A subsequent study evaluated the same therapy in the experimental autoimmune uveitis (EAU) model induced by interphotoreceptor retinoid-binding protein (IRBP) peptide in B10.RIII mice (Ref. 38). Animals pre-treated with the therapy intravitreally exhibited improved clinical signs of intraocular inflammation and reduced expression of proinflammatory markers. Safety assessments in healthy controls showed no changes in retinal thickness and function, suggesting the therapy's safety for intraocular delivery (Ref. 38). EAU is a T cell-mediated intraocular inflammation model that resembles the human autoimmune condition and is suitable for testing new therapies for chronic posterior uveitis, a disease that is particularly challenging to treat (Ref. 5).

It is important to highlight that investigating gene therapies for uveitis in two distinct experimental models is a suitable approach given the multifactorial nature of the disease and the difficulty in mimicking all aspects of the human condition. In fact, no single model can recapitulate all features of human uveitis; each one is unique and exhibits specific characteristics of the disease (Ref. 5). Ridley *et al*. (Ref. 38) expanded on the findings reported in the EIU model described by Ildefonso *et al*. (Ref. 95) by employing an experimental model that more accurately recapitulates features of human uveitis and conducted clinical examinations commonly used in human disease monitoring. Nonetheless, a very thorough safety evaluation should indeed be considered to assess the effects of constitutive expression of a viral protein in the eye, not least because it is known that the introduction of exogenous viral proteins in the eye can trigger immune responses and actually exacerbate inflammation (Ref. 96). Therefore, the use of multiple uveitis models is desirable and might provide additional insights into mechanisms, safety and efficacy and facilitate the screening of potential therapeutic candidates for clinical application.

For instance, Lo *et al*. explored the specificities of two types of EAU to study the role of Toll-like receptors (TLRs) in uveitis development and propose a gene therapy approach (Ref. 37). The EAU induced by IRBP immunisation with adjuvants and the model established by adoptive transfer of IRBP-specific T cells were employed in order to comprehend responses not triggered by adjuvants and select a target involved in both models. A gene therapy consisting of AAV8 encoding short hairpin RNA silencing TLR7 was thus pursued once TLR7 signalling activation led to impairment of the barrier function of retinal pigment epithelium (RPE) cells and exacerbation of the inflammatory response (Ref. 37). TLR7 was upregulated in both disease models and subretinal treatment with its agonist led to exacerbation of the inflammatory response in EAU mice (Ref. 37). A subretinal injection with this therapy given to mice on the day of uveitis induction resulted in attenuated disease severity in both models 12 days later. This preliminary data confirmed the involvement of TLR7 signalling in EAU development and may support further studies targeting this pathway for uveitis. In addition, the authors drew important conclusions regarding mechanisms implicated in TLR7 signalling in EAU with the proposed therapy. Hence, the study underscores the applicability of AAV-mediated gene delivery both as a therapeutic approach and as a means to enhance our understanding of disease pathogenesis. Moreover, RNA-mediated silencing is a promising approach for ocular diseases and has shown encouraging results in clinical trials (Ref. 97). This strategy, combined with AAV-mediated delivery and expression using cell-specific promoters, may reduce the risk of off-target effects, a common concern in RNA interference, and improve the safety of gene therapy candidates (Ref. 97).

#### Gene therapies assessed in multiple disease models

Ildefonso *et al*. reported a different therapeutic strategy and assessed the therapy in multiple disease models (Ref. 98). The authors utilised NaIO_3_-induced RPE oxidative injury and EIU models to investigate the anti-inflammatory and antioxidative properties of a peptide derived from the Nrf2 (Ref. 98). The Nrf2-derived peptide was fused to the Tat sequence to promote cell penetration of the expressed protein, which was validated in the study (Ref. 98). The secreted and cell-penetrating peptide allows the nuclear translocation of endogenous Nrf2, whose signalling seems to be involved in both neuroprotection effective in glaucoma models and the pathogenesis of uveitis. This peptide sequence was packaged into AAV2 (quadY-F + T-V), the same mutant used in Ildefonso *et al*. (Ref. 95), and intravitreally injected into mice prior to disease induction (Refs 95, 98). Reduced levels of proinflammatory cytokines and fewer infiltrating cells were reported in animals with acute oxidative stress damage and uveitis, respectively. Additionally, reduced expression of a marker for protein oxidation and improved retinal function were reported in the oxidative stress model, although it did not result in complete protection of RPE cells as showed in morphological analysis (Ref. 98). Despite these positive findings, the authors drew attention to the potential risk of ocular infection with continuous inflammatory suppression in the eye, which could be attenuated with a controlled expression of anti-inflammatory factors. Still, by targeting both oxidative stress and inflammation, this therapy affects processes mutually implicated in the pathogenesis of uveitis and glaucoma, as well as other ocular diseases, and could potentially benefit patients with hard-to-treat chronic conditions.

Similarly, Li *et al*. developed a gene therapy targeting ocular inflammation and choroidal neovascularisation (CNV) and assessed its efficacy in EAU, EIU and laser-induced CNV models (Ref. 36). The treatment consisted of three inhibitors: one that binds to vascular endothelial growth factor A (VEGFA), the other to C3b/C4b components of complement and a dual inhibitor containing both VEGF and complement binding motifs. The construct was packaged and intravitreally delivered to mice before disease induction. A variant of AAV2 with six mutations (sext Y-F; Y252, 272, 444, 500, 704, 730F) was used for widespread retinal transduction. The therapy ameliorated inflammation and neovascularisation in all tested models, with the dual-acting system promoting better results in EAU and CNV but not in EIU, compared with each inhibitor alone (Ref. 36). Interestingly, only the combined and the anticomplement alone approaches reduced inflammatory infiltration in EIU, whereas the three strategies improved EAU, highlighting the differences in these experimental mechanisms and supporting the implication of VEGF and the complement cascade in EAU pathogenesis (Ref. 36). This result is promising, knowing that elevated levels of VEGF have been detected in the vitreous and aqueous humour of patients with different types of uveitis and it could be a causative factor in uveitic macular oedema (Refs 99, 100, 101). In addition, uveitis and uveitic macular oedema can lead to retinal and choroidal neovascularisation, complications usually treated with anti-VEGF therapy (Refs 102, 103). Therefore, these findings brought attention to what may be possible with multigenic constructs as alternatives for uveitis treatment. The study also reinforced the value of testing new drug candidates in multiple models of uveitis as therapeutic effects seen in EAU were not replicated in EIU.

#### Gene therapies assessed in a single uveitis model

Kumar *et al*. also explored the contribution of complement activation to uveitis pathogenesis and suggested AAV2-mediated delivery of soluble CD59 (sCD59) as a potential gene therapy (Ref. 104). CD59 prevents the formation of the membrane attack complex (MAC), which seems to be associated with the activation of nucleotide-binding domain-like receptors 3 (NLRP3) inflammasome, involved in several inflammatory conditions. Intravitreal injection of this therapy was given to mice before EAU induction by IRBP. It resulted in attenuated inflammation, with worsened disease severity and progression found in control subjects. However, this therapeutic effect seems to be independent of MAC formation since similar results were not seen in transgenic mice unable to assemble MAC subjected to the same disease model (Ref. 104). Still, the gene therapy inhibited MAC deposition and the subsequent NLRP3-mediated production of IL-1β in EAU retinas, which was also seen in transgenic animals. The authors bring attention to the variability of disease severity observed with the animal model employed, which might have contributed to the unexpected outcomes. In addition, they report other mechanisms that might be involved in CD59 anti-inflammatory effects in EAU.

Recent reports from clinical trials conducted by the same group using AAV2-sCD59 (named JNJ-1887) for wet age-related macular degeneration and geographic atrophy (NCT03144999; NCT03585556) are encouraging. These findings may pave the way for its potential application in NIU should further studies prove its safety and efficacy (Ref. 105). It is important to mention that control animals treated with AAV2-GFP exhibited higher clinical scores, albeit not statistically significant, compared with the placebo phosphate buffer-treated group, indicating a possible inflammatory response associated with the vector or the reporter. This finding is in line with reports from the first clinical trial with JNJ-1887 (formerly named HMR59), in which CS therapy was required to control inflammation in a significant number of patients (Ref. 106). In fact, gene therapy-associated uveitis is a condition previously reported in clinical and preclinical studies, with its potential cause attributed to vector dose (Refs 107, 108). This dose-dependent inflammation has also been observed in non-ocular gene therapies, representing a common concern in the field since it can affect efficacy and lead to toxicity (Ref. 107). This issue is especially relevant when assessing AAV-mediated gene therapies for inflammatory conditions, such as uveitis, whose clinical assessment is likely to be misinterpreted by a vector-related reaction. Therefore, efforts have been made to develop engineered vectors with lower immunogenicity, as well as to establish better immunomodulation protocols and to optimise manufacturing processes to avoid immunogenic impurities (Ref. 109). Additionally, engineered vectors with enhanced tropism may allow for lower doses and consequently reduce the risk of vector-related inflammatory reactions (Ref. 110). Indeed, multiple AAV serotypes with a variety of mutations have been employed in recent investigations for uveitis.

For example, Qiu *et al*. (Ref. 112) used a tyrosine-capsid mutant (Y733F) AAV8, which has previously demonstrated higher and faster transduction efficiency in murine retinal cells compared with wild-type AAV8 (Refs 111, 112). The vector has additionally showed efficient transduction in RPE and photoreceptor layers after subretinal delivery in mice (Ref. 112). Hence, the authors chose the subretinal route, which is known to induce a milder inflammatory response compared with the intravitreal (Ref. 113). These vectors were used to deliver the angiotensin-converting enzyme 2 (ACE2) gene, which has shown beneficial effects in models of glaucoma and diabetic retinopathy because of its ability to counteract the deleterious effects of angiotensin II and thereby attenuate inflammation and fibrosis (Refs 112, 114). Subretinal treatment with the therapy given before EAU induction by IRBP resulted in clinical and functional improvement (Ref. 112). It also decreased the expression of inflammatory cytokines and inhibited pathways involved in the pathological process of EAU. Therefore, the study highlights the importance of the renin–angiotensin system (RAS) in regulating ocular inflammation and provides a novel approach by targeting the ACE2/Ang-(1–7)/Mas protective axis. However, Qiu *et al*. (Ref. 112) primarily demonstrated the protective effect of the gene therapy administered prior to insult/disease, which is consistent with most studies discussed in the review. Although a protective effect may be applicable for managing recurrent uveitis, an ideal therapeutic approach should additionally guarantee efficacy if the therapy is administered following the onset of the disease. Further investigation addressing the vector's efficiency in ex vivo/in vitro human models seems to be a logical way to assist in documenting the impact of the timing of administration. Hickey *et al*. reported limited transduction efficiency, mainly restricted to the inner nuclear layer, in degenerated human explants treated with the same vector, AAV8 (Y733F) (Ref. 63). In contrast, better results were seen in healthy explants from primates and in mice with retinal degeneration treated with subretinal and intravitreal injections (Ref. 63). The disparity in AAV transduction efficiency across different species has been discussed in several studies, highlighting the importance of screening vectors in multiple settings (Refs 115, 116, 117, 118). Moreover, it brings attention to the value of human explants in ocular gene therapy development owing to their resemblance to in vivo conditions and suitability for translational research (Ref. 119).

Notably, the studies targeting uveitis discussed thus far in this review did not include an experimental group treated with CS or conventional immunosuppressive drugs as positive controls. It does not affect the scientific contribution of each study, although knowing the performance of such therapies compared with conventional drugs might maximise their clinical prospects. On the other hand, Crabtree *et al*. tested their developed gene therapy against topical CS. The gene therapy consisted of scAAV8 vectors encoding the codon-optimised human leucocyte antigen-G (HLA-G) isoforms 1 and 5 (Ref. 34). HLA-G is involved in maintaining the ocular immune privilege and has shown beneficial effects in models of dry eye, corneal inflammation and neovascularisation (Ref. 18). An intravitreal injection containing equal amounts of HLA-G1 and HLA-G5 packaged into scAAV8 was given to rats before EAU induction by IRBP. AAV-mediated expression of HLA-G1/5 led to attenuated inflammation, but dexamethasone-treated animals exhibited better efficacy. However, topical CS treatment was accompanied by severe systemic side effects (Ref. 34). Therefore, the study did demonstrate the advantage on side effects of long-lasting expression of HLA-G1/5 over standard therapy with multiple instillations of CS in a model of uveitis. Although the biodistribution study did not suggest systemic expression of HLA-G1/5, even with neutralising antibodies (Nabs) against AAV8 found in the serum, a more targeted and controlled production would warrant additional safety for this therapy given the tumorigenic potential of HLA-G proteins (Ref. 120). Also, the use of AAV8 for intravitreal delivery is encouraged because of the lower prevalence of Nabs against this serotype compared with commonly used vectors found in human vitreous (Ref. 121). However, the low retinal tropism of AAV8 through this administration route should be considered in models such as EAU, in which the inflammation involves both the anterior and posterior segments of the eye (Refs 5, 122, 123). Therefore, capsid modifications leading to improved retinal tropism, as reported with tyrosine-to-phenylalanine mutations, may be advantageous in future studies with this therapy (Ref. 111).

### Current progress and future directions

**Figure 2.**
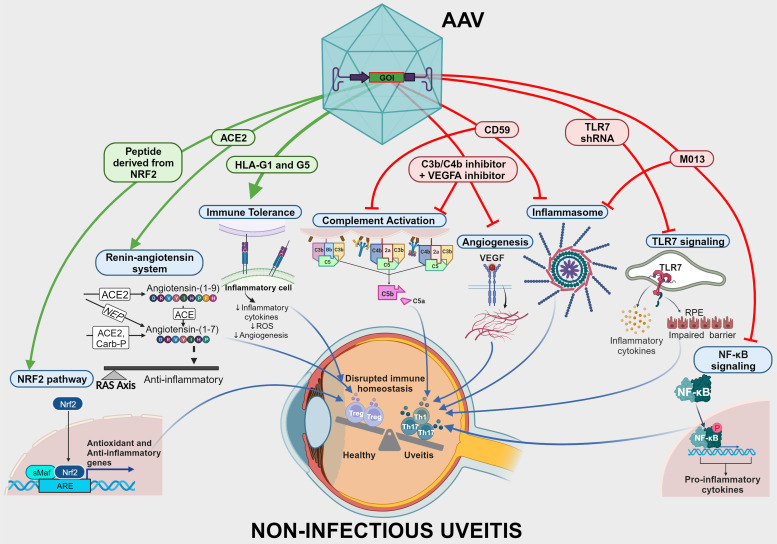
Overview of delivered genes and molecular targets of recent preclinical studies with AAV-mediated gene therapies for the treatment of NIU. AAV, adeno-associated virus; ACE2, angiotensin-converting enzyme 2; ARE, antioxidant response element; GOI, gene of interest; HLA-G, human leucocyte antigen-G; NEP, neprilysin; NF-κB, nuclear factor kappa B; NIU, non-infectious uveitis; Nrf2, nuclear factor erythroid factor 2-related factor 2; RAS, renin–angiotensin system; shRNA, short hairpin RNA; TLR7, Toll-like receptor 7; VEGFA, vascular endothelial growth factor A.

AAV-mediated gene expression may offer a local and long-lasting therapy alternative for uveitis patients, improving patients' adherence and alleviating the treatment burden. It may also allow efficient control of the inflammatory response while it reduces risks of undesirable off-target effects with local delivery. Altogether, the studies reviewed here explored multiple pathways implicated in ocular inflammation (Fig. 2) and undoubtedly proposed biologically credible AAV-mediated gene therapies for NIU. They also addressed a variety of strategies that may be employed in AAV-based platforms to ensure optimised gene delivery and therapeutic effect (Table 4). However, further research assessing these therapies when administered after disease induction is needed to accurately represent efficacy in the practical clinical scenario as well as to improve the understanding of the impact of disease-triggered inflammatory responses on vector's efficiency. Furthermore, given the risk of systemic immunosuppression and opportunistic infections, tight expression control and vector specificity are important features to be considered in such therapies. As a multifactorial disease, uveitis has a complex pathogenesis that is not fully understood. In-depth knowledge of animal models and progresses in ocular immunology may contribute to the identification of additional targets for future gene therapies for uveitis and other ocular inflammatory diseases. The diversity of pathways involved in NIU pathogenesis also encourages the development of dual-acting gene therapies targeting multiple processes. A growing number of clinical trials with gene therapies for ophthalmic conditions is likely to advance our understanding of vector-induced inflammation and facilitate the development of effective strategies to control and prevent it. This would not only positively impact uveitis gene therapy research but could potentially boost the relatively limited number of studies utilising AAV-based platforms for uveitis, ultimately paving the way for clinical translation.

**Table 4.** Strategies and main outcomes of preclinical studies using AAV-mediated gene therapies in models of uveitis

Vector-related strategy	Construct-related strategy	Main outcomes	Ref.
Engineered AAV2 with five mutations (quadY-F + T-V) for enhanced retinal transduction after intravitreal delivery	Insertion of a cell-penetrating peptide sequence upstream of the transgene. Constitutive expression	It decreased concentration of IL-1β in the vitreous and reduced inflammatory infiltrate	95
Wild-type serotype with high transduction efficiency in RPE cells after subretinal delivery (AAV8)	RNA-mediated gene silencing	Decrease of inflammation scores, improvement of morphology and reduction of inflammatory infiltrate and cytokines	37
Engineered AAV2 with five mutations (quadY-F + T-V) for enhanced retinal transduction after intravitreal delivery	Insertion of a cell-penetrating peptide sequence upstream of the transgene. Codon optimisation and constitutive expression	It increased expression of antioxidant genes in healthy animals. Improvement of retinal function, and reduction of levels of inflammatory cytokines and protein oxidation marker in the oxidative stress model. It did not prevent damage in RPE cells. Reduction of inflammatory infiltrate in the uveitis model	98
Engineered AAV2 with six mutations (sext Y-F) for enhanced retinal transduction after intravitreal delivery	Multigenic dual-acting therapy. Constitutive expression	Reduction of infiltrating cells’ number in EIU model for combined and anticomplement therapies. Combined, anti-VEGF and anticomplement therapies improved histopathological and clinical changes in EAU and CNV. The combined therapy outperformed the others in all models	36
Wild-type serotype with wide retinal transduction (AAV2)	Deletion of GPI anchor to enable secretion of the protein	It decreased MAC deposition and NLRP3-mediated IL-1β production. Preservation of retinal function and reduction of cell infiltration and structural damage	104
Engineered AAV8 with 1 mutation (Y733F) for enhanced RPE and photoreceptors transduction	Constitutive expression	Attenuation of inflammation with decreased clinical and histological score. Improvement of retinal function and reduction of expression of inflammatory cytokines	112
Self-complementary vector for faster transgene expression; wild-type serotype with weak overall retinal transduction after intravitreal delivery (AAV8)	Codon optimisation and constitutive expression	Reduction of clinical and histological scores, similar to corticosteroid treatment. Systemic side-effects for corticosteroid-treated group. Absence of viral genome in other organs but detection of Nabs in the serum	34

AAV, adeno-associated virus; CNV, choroidal neovascularisation; EAU, experimental autoimmune uveitis; EIU, endotoxin-induced uveitis; F, phenylalanine; IL-1β, interleukin 1β; MAC, membrane attack complex; Nabs, neutralising antibodies; RPE, retinal pigment epithelium; T, threonine; V, valine; VEGF, vascular endothelial growth factor; Y, tyrosine; GPI, glycosylphosphatidylinositol.

## Concluding remark

Glaucoma and uveitis are sight-threatening diseases with complex pathogenesis that necessitate improved therapeutic alternatives because of unmet clinical need, compliance challenges and side effects of current treatments. AAV-mediated gene therapies emerge as a promising approach to overcome these challenges, offering a potential solution to the complexities associated with treating these conditions. Clinical trials have reported positive outcomes employing this technology to treat other acquired ocular diseases, which, despite being primarily vascular conditions, share many of the underlying pathological processes of glaucoma and uveitis. Already, multiple strategies and targets have been explored in preclinical studies for glaucoma and uveitis, demonstrating encouraging results regarding safety and efficacy.

In glaucoma, preclinical studies targeting neuroprotection have attempted to overcome limitations regarding long-term expression of the neurotrophic factor BDNF and safety issues from off-target effects of antiapoptotic proteins. In this regard, strategies such as multigenic constructs and cell-specific promoters were employed and have demonstrated positive results. Other approaches explored different pathways providing neuroprotection to RGCs, such as Nrf2 and the tumour suppressor gene MYC signalling, the first controlled by a physiologically induced promoter. Moreover, findings reported with gene therapies affecting other processes involved in the disease mechanism such as oxidative stress, inflammation and the aqueous humour outflow have led to important discussion about treatment timing and use of engineered and self-complementary vectors. These studies have incorporated advanced technologies for gene expression optimisation such as gene editing and drug-inducible systems.

In preclinical studies for uveitis, diverse experimental strategies have highlighted the importance of assessing gene therapies in multiple uveitis and disease models. The first approach was used to investigate therapies targeting the inflammasome, NF-κB- and TLR pathways. In addition, these studies employed relevant therapeutic/delivery strategies, such as engineered vectors, RNA interference and insertion of cell-penetrating sequences. These interventions not only mitigated inflammation under the tested conditions but also contributed to an in-depth understanding of uveitis mechanisms. The utilisation of multiple disease models proved advantageous, especially when studying a gene with dual-acting effects or a multigenic cassette, leading to positive outcomes in combination with vector optimisation. However, concerns about infection susceptibility under continuous immunosuppression emphasise the benefits of controlled expression systems in uveitis gene therapies. Lastly, therapies assessed in a single model of uveitis targeting the complement system, RAS axis or immunotolerance have prompted discussions about viral tropism in different administration routes, gene therapy-associated uveitis, the importance of positive control groups and the necessity to evaluate AAV-based platforms in diverse species, including human settings.

Therefore, careful consideration regarding experimental designs and disease models is crucial to adequately evaluate novel gene therapies for glaucoma and uveitis and enhance the potential for clinical translation. Hence, future directions should focus on further refining AAV-mediated gene delivery and expression, including engineered vectors, optimised constructs and new targets, alongside obtaining an in-depth understanding of disease mechanisms and models.
